# Biofilm formation of *Clostridium difficile* and susceptibility to Manuka Honey

**DOI:** 10.1186/1472-6882-14-329

**Published:** 2014-09-03

**Authors:** Eric N Hammond, Eric S Donkor, Charles A Brown

**Affiliations:** Department of Microbiology, University of Wales Institute Cardiff, Cardiff, CF1 3TL UK; Department of Microbiology, University of Ghana Medical School, Accra, Ghana; Department of Medical Laboratory Sciences, School of Allied Health Sciences, University of Ghana, Accra, Ghana

**Keywords:** *Clostridium difficile*, Biofilm, Manuka honey, Antibacterial, Susceptibility

## Abstract

**Background:**

Biofilm bacteria are relatively more resistant to antibiotics. The escalating trend of antibiotic resistance higlights the need for evaluating alternative potential therapeutic agents with antibacterial properties. The use of honey for treating microbial infections dates back to ancient times, though antimicrobial properties of Manuka honey was discovered recently. The aim of this study was to demonstrate biofilm formation of specific *Clostridium difficile* strains and evaluate susceptibility of the biofilm to Manuka honey.

**Methods:**

Three *C. difficile* strains were used in the study including the ATCC 9689 strain, a ribotype 027 strain and a ribotype 106 strain. Each test strain was grown in sterile microtitre plates and incubated at 37°C for 24 and 48 hours in an anaerobic cabinet to allow formation of adherent growth (biofilm) on the walls of the wells. The effect of Manuka honey on the biofilms formed was investigated at varying concentrations of 1-50% (w/v) of Manuka honey.

**Results:**

The three *C. difficile* strains tested formed biofilms after 24 hours with the ribotype 027 strain producing the most extensive growth. There was no significant difference (p > 0.05) found between the amount of biofilms formed after 24 and 48 hours of incubation for each of the three *C. difficile* strains. A dose–response relationship between concentration of Manuka honey and biofilm formation was observed for all the test strains, and the optimum Manuka honey activity occurred at 40-50% (v/v).

**Conclusion:**

Manuka honey has antibacterial properties capable of inhibiting *in vitro* biofilm formed by *C. difficile*.

## Background

Biofilms are complex structures of polysaccharide matrix excreted by bacteria in a form of slimy, glue-like substance that adhere to material surfaces especially, when exposed to some amount of water [1]. Within the last decade, many studies have been done on biofilms which have established their association with infections and contaminations [1]. The conventional methods of killing bacteria by using antibiotics and disinfection are often unsuccessful with biofilm forming bacteria [1]. Thus biofilm forming bacteria may pose a relatively greater threat to public health. For instance, it is reported that patients suffering from biofilm associated infections tend to stay longer in hospital than expected [2, 3]. Worldwide, it is estimated that biofilms are associated with 65 percent of nosocomial infections and contribute to high death rate and 2-14% of all surgical wounds complications [2]. Biofilm formation by bacteria also has serious economic implications. It is reported that a microorganism associated with biofilm formation is reported to cost some nations billions of dollars yearly in medical infections, equipment damage and product contamination [4].

Some bacterial organisms known to form biofilms include *Pseudomonas aeruginosa, Staphylococcus aureus, Listeria monocytogenes* and *Clostridium difficile* [5, 6]. Biofilm formation by *C. difficile* was first reported in 2012 and has since then been demonstrated with a few strains of the organism [7–10]. Evidence from some studies indicate that amount of biofilm formed by *C. difficile* varies from strain to strain [4, 5]. Mutagenesis studies have identified several surface proteins such as SlecC, Cwp84 and LuxS that are required for biofilm formation of *C. difficile* [8, 10]. There is also evidence that some level of sporulation occurs in *C. difficile* biofilm [11, 12].

The use of honey for treating microbial infections dates back to ancient times, though antimicrobial properties of Manuka honey was discovered recently. The antibacterial effect of Manuka honey against bacterial biofilms has been demonstrated for several organisms such as *Streptococcus pyogenes* [13], *Pseudomonas aeruginosa* [14], *Enterococcus faecalis* [15] and *Streptococcus mutans* [16]. Bactericidal effects have been found in both planktonic cultures and biofilm, although higher concentrations were required to inhibit biofilms [17]. In a biofilm study, Maddocks *et al*. [17] reported that sublethal concentrations of Manuka honey disrupted the binding of *S. pyogenes* to the human fibronectin but did not prevent binding to fibrinogen. Manuka honey thus appears to be a promising antibacterial agent in this era of diminishing antimicrobial agents. However, further studies on its antibacterial properties are required especially, with highly resistant pathogens such as *C. difficile*.

*C. difficile,* the causative agent of severe inflammation of the bowel (pseudomembranous colitis), has become the most significant nosocomial antibiotic-associated diarrhoea reported worldwide [18, 19]. The Centers for Disease Control and Prevention (CDC) estimate that nearly 250,000 serious C. difficile infections (CDI) occur in the US annually, at a cost of at least one billion dollars, resulting in 14,000 deaths (CDC, 2013) [20]. This high public health burden associated with *C. difficile* is partly due to the trend of increasing resistance of the organism to several essential antibiotics, a problem which highlights the need for alternative treatment methods of *C. difficile* infections. In a previous study, we demonstrated the susceptibility of *C. difficile* to Manuka honey (*Leptospermum scoparium*) [21]. The findings of the study showed that Manuka honey exhibits a bactericidal activity against *C. difficile* with minimum inhibitory and bactericidal concentrations of 6.25% (v/v) [21]. However, it is unknown if biofilm formed by *C. difficle* is also susceptible to Manuka honey. Biofilm formation by *C. difficile* in itself has been recently reported, and it is important to confirm this with various *C. difficile* strains. The aim of this study was to demonstrate biofilm formation of specific strains of *C. difficile* and the antibacterial effect of Manuka honey on the *C. difficile* biofilm.

## Methods

### *Clostridium difficile*strains

Three *C. difficile* strains were used for the experiments in this study, and they included the ATCC 9689 strain, a ribotype 027 strain and a ribotype 106 strain. These strains were selected for the study due to their epidemiological or clinical significance. The *C. difficile* strains were obtained from University of Wales Hospital and were maintained in Robertson’s Cook meat medium (Oxoid, Cambridge, UK) at the Department of Microbiology, University of Wales Institute Cardiff where the study was carried out. Prior to using the *C. difficile* strains in the experimental work, they were purified on blood agar plates.

### Manuka honey

Woundcare™ 18+ Active Manuka honey (potency equivalent of greater than 18% (w/v) phenol) with non-peroxide antibacterial activity from Comvita UK was used in this study.

### Microtitre plate assay for the assessment of biofilm formation in C. difficile strains

The experiments performed to determine the capability of the *C. difficile* strains to form biofilms were based on the previously described methods [4]. The three *C. difficile* test strains were cultured overnight in Reinforced Clostridial Medium (RCM) broth at 37°C for 24 hours. For each strain, a dilution of 1:100 inoculum was made in a sterile broth bottle by pipetting 1 ml of each strain into 99 ml of RCM broth and vortexing to achieve a good mixture. An aliquot of 200 μl of each diluted inoculum was dispensed into a 96-well Nunc flat bottom microtitre plate. The plates and contents were incubated at 37°C for 24 and 48 hours in an anaerobic cabinet to allow for formation of biofilms on the walls of the wells. For each experiment, wells of RCM broth without *C. difficile* strains were used as negative control. At the end of the 24 and 48 hours of incubation, the plates were removed from the anaerobic cabinet and the cultures were carefully removed by using a Pasteur pipette. Subsequently, 200 μl of 2.5% glutaraldehyde solution was pipetted into each of the drained wells, and allowed to stand for 5 minutes to allow fixation. The glutaraldehyde solution was then removed and the empty wells were washed by dispensing 200 μl of phosphate buffered saline (PBS) (Oxoid, Cambridge, UK) in them. The PBS was discarded and the wells were stained with 200 μl of 0.25% (w/v) aqueous crystal violet for 5 minutes. After this time, the wells were washed with PBS eight times and allowed to air dry. The quantity of biofilm formed was analyzed by adding 200 μl solvent (1:1 ethanol and acetone solution) to each well to dissolve dye from adherent cells (biofilm). Absorbance was measured within 5 minutes of adding the solvent at 570 nm using a Dynex plate reader. The microtitre plate biofilm assay was performed three times on separate occasions for all test *C. difficile* strains.

### Determination of the effect of various honey concentrations on C. difficile biofilms

The three *C. difficile* test strains were cultured overnight in an RCM broth at 37°C for 24 hours. For each strain, an aliquot of 200 μl of each diluted inoculum (as described in Section 3.3) was dispensed into each well of a 96-microtitre plate, starting from the first column (Column 1) which contained inoculum but no Manuka honey (positive control). The last column (Column 12) contained RCM only (no inoculum) and was used as a negative control. Outer rows and columns were unused, except for column 12 to avoid edge effects. The plates were incubated at 37°C for 24 hours in an anaerobic cabinet to allow for the formation of biofilms on the walls of the wells. After incubation, a sterile Pasteur pipette was used to carefully remove the liquid phase containing any planktonic growth from each well into a discard jar, leaving adherent biofilm attached to the well walls. However the positive control was re-filled with 200 μl RCM to keep the biofilm alive. With the exception the negative control wells, each well was re-filled with 200 μl of Manuka honey solutions and incubated at 37°C for 24 hours in an anaerobic cabinet. Different concentrations of Manuka honey (0, 1, 2, 4, 8, 10, 20, 30, 40, 50% (w/v)) were included in the experimental setup and were prepared by dilutions with RCM.

After incubation, the liquid phase in each well (containing any overnight planktonic growth together with the honey solutions) was discarded into the discard jar using a sterile Pasteur pipette. The adherent growth was fixed with 200 μl of 2.5% glutaraldehyde for 5 minutes. The fixative was removed into a toxic waste bottle and the wells washed twice with PBS. All the wells were stained with 200 μl of 0.25% crystal violet for 5 minutes. The stain was then removed and the wells washed 8 times with PBS. It was ensured that any remaining liquid was drained from the plates by inverting them vigorously onto paper hand towels. At this point, biofilms were visible as purple rings formed on the side of each well. The quantity of biofilms formed were analysed by adding 200 μl solvent (1:1 ethanol and acetone solution) to each well to dissolve dye associated cells. The reading was taken (within 5 minutes of adding the solvent) at 570 nm in a Dynex plate reader. The microtitre plate biofilm assay was performed three times on separate occasions for all *C. difficile* test strains.

### Statistical analysis

The experimental data was entered into Microsoft Excel® 2010 and analyzed. Mean biofilm biomass was calculated for each *C. difficile* strain at 24 and 48 hours. The Student's *t*-test was used to test for any significant difference between biofilms formation as function of the hours for the different strains. A *p*- value less than or equal to 0.05 was considered significant.

## Results and discussion

**Figure 1:**
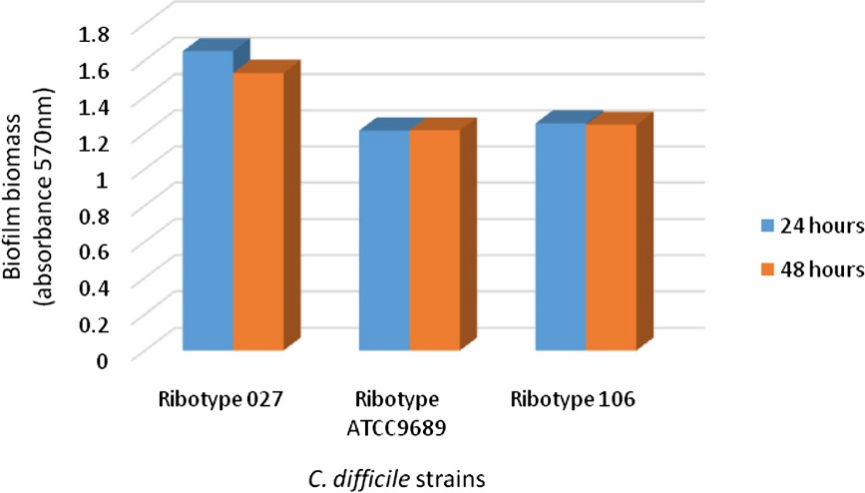
**Formation of adherent growth (biofilm) by** ***C. difficile*** **strains after 24 and 48 hours incubation.**

In this study we investigated biofilm formation of *C. difficile* and the susceptibility to Manuka honey. Figure 1 shows that all the three *C. difficile* strains formed biofilms and there was no significant difference (*p* = 0.3901) between biofilm formation in 24 hours and biofilm formation in 48 hours.

Bacterial biofilm formation occurs through an interesting mechanism which first involves bacterial attachment to a material surface. This initial attachment of the bacterial organism is influenced mainly by combination of environmental factors (such as nutrient levels, temperature, pH and duration of attachment) and genetic factors [1]. Immediately the cells are attached to suitable surfaces, they begin to multiply and grow to form a thin layer (monolayer) towards where conditions are favourable on material surfaces. At this stage, the cells undergo a developmental change which lead to the production of a complex structure referred to as exopolysaccharide glycocalyx polymers matrix and is one of the hallmarks of a matured biofilm [22–24].

The ability of *C. difficile* to form biofilms as observed in this study concurs with studies carried out by several other investigators [7–10]. As shown in Figure 1, among the three *C. difficile* strains, the ribotype 027 strain showed the highest potential for biofilm formation. Biofilm formation has been linked with virulence of several bacterial pathogens including *C. difficile* [8, 13]. *C. difficile* of ribotype 027 is known to be a hypervirulent strain and the commonest cause of *C. difficile* associated outbreaks [25]. Data from our study appear to indicate that the high virulence and epidemiological significance of *C. difficile* ribotype 027 strains may be related to its relatively greater ability to form biofilms compared to other *C. difficile* strains such as ribotype 106 strains. In their study of *C. difficile* biofilm, Dapa *et al*. [8] also observed that the hypervirulent strain (ribotype 027) produced more biofilm than a less virulent *C. difficile* strain. Currently, very little is known about the actual role of biofilm in the pathogenicity and pathogenesis of *C. difficile*, and further studies are required to elucidate this.

**Figure 2:**
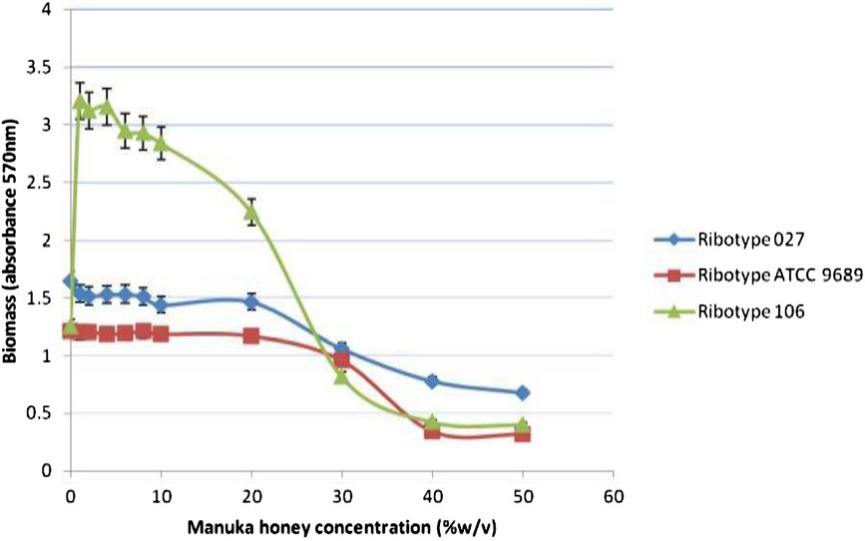
**The effect of varying concentrations of Manuka honey on biofilm of** ***C. difficile*** **strains.**

The extent of Manuka honey inhibiting the established biofilm was determined by comparing the amount of biofilm formed in the wells with and without Manuka honey. This experiment showed that generally, there was a dose–response relationship between the amount of biofilm depleted and concentration of Manuka honey (Figure 2). Concentrations of Manuka honey below 20% (w/v) appeared to have no or little effect on the established biofilm for each of the three *C. difficle* strains. However, concentrations between 20 and 50% (w/v) Manuka honey resulted in decreasing amount of biofilm formed by all test strains after 24 hours. Although MIC and MBC of Manuka honey against suspensions of the *C. difficile* strains used in this study were 6.25% (v/v) [21], much higher concentrations of 30-50% (w/v) of Manuka honey were required to deplete biofilms formed by the *C. difficile* strains.

This may be due to the ability of the sessile bacteria (biofilm formed by *C. difficile*) to secrete proteins and polymeric sugars which serve as a protection to enhance quorum development for survival [1]. Similar results were reported by Okhiria *et al*. [26]. Fux *et al*. [27] showed that biofilms are strongly resistant to biocides, drying and most environmental stresses. Ashby *et al.* [28] and Costerton *et al.* [29] reported that the ability of biofilm to resist antibiotic effect could be due to the slow growth rate of biofilm, since the effect of bactericides on biofilm usually declines with lower growth rate.

From this study, it may be inferred that the most suitable Manuka honey concentrations to inhibit *C. difficile* biofilm significantly were 40 and 50% (w/v). Similarly, Okhiria *et al*. [26] reported that *Pseudomonas* biofilm exposed to 40% (w/v) Manuka honey concentration showed a significant inhibition, but 20% (w/v) Manuka honey did not show any significant inhibition. From Figure 2, it can be observed that Manuka honey could not exhibit 100% depletion of biofilm formed by the *C. difficile* strains. Studies by Alandejani *et al*. [30] also reported that the effectiveness of Manuka honey against biofilms formed by *S. aureus* and *P. aeruginosa* were less than 100%. Nevertheless, based on this study and others, it is important to note that Manuka honey has an appreciable antibacterial activity against biofilm formed by bacterial organisms [31]. While in this study we demonstrated the ability of Manuka honey to inhibit biofilm formation of *C. difficile*, Maddock *et al*. [17] demonstrated the ability of Manuka honey to disrupt pre-formed biofilms of *Streptococcus pyogenes*. Recently, Ansari *et al*. [31] also reported that Jujube honey can disrupt pre-formed biofilms of *Candida albicans*. Antimicrobial effect of Manuka honey is due to a property referred to as Unique Manuka Factor that is absent in other types of honey [32]. Various studies have revealed that the active ingredient in Manuka honey is Methylglyoxal [33, 34], and this compound is known to have synergistic effect with some antibiotics such as piperacillin [35].

We previously demonstrated susceptibility of *C. difficile* to Manuka honey, and in this study, have also shown that biofilm formed of the organism is similarly susceptible to Manuka honey. Overall these findings have important applications in the treatment of *C. difficile* infections given the escalating trend of the organism to several essential antibiotics. In the light of the findings of the current study, it is also important for further studies to determine the rate and concentrations at which Manuka honey inhibits biofilms of *C. difficile in vivo*. Additionally, it would useful to investigate the effect of Manuka honey on spores of *C. difficile*.

## Conclusions

In this study, we have demonstrated biofilm formation of specific *C. difficile* strains including ATCC 9689, ribotype 027 and ribotype 106. We have also shown that Manuka honey exhibits antibacterial activity against *C. difficile* biofilm with the optimum activity occurring at 40-50% (w/v). The bactericidal action of Manuka honey may be exploited practically by incorporating a solution of 40-50% (w/v) Manuka honey in topical and hand-washing formulations in care homes and hospitals or places where *C. difficile* populations are likely to be high.
